# An analysis of *Echinacea* chloroplast genomes: Implications for future botanical identification

**DOI:** 10.1038/s41598-017-00321-6

**Published:** 2017-03-16

**Authors:** Ning Zhang, David L. Erickson, Padmini Ramachandran, Andrea R. Ottesen, Ruth E. Timme, Vicki A. Funk, Yan Luo, Sara M. Handy

**Affiliations:** 1 Center for Food Safety and Applied Nutrition, Office of Regulatory Science, US Food and Drug Administration, College Park, Maryland 20740 United States; 2 Department of Botany, National Museum of Natural History, MRC-166, Smithsonian Institution, PO Box 37012, Washington, DC 20013-0166 USA

## Abstract

*Echinacea* is a common botanical used in dietary supplements, primarily to treat upper respiratory tract infections and to support immune function. There are currently thought to be nine species in the genus *Echinacea*. Due to very low molecular divergence among sister species, traditional DNA barcoding has not been successful for differentiation of *Echinacea* species. Here, we present the use of full chloroplast genomes to distinguish between all 9 reported species. Total DNA was extracted from specimens stored at the National Museum of Natural History, Smithsonian Institution, which had been collected from the wild with species identification documented by experts in the field. We used Next Generation Sequencing (NGS) and CLC Genomics Workbench to assemble complete chloroplast genomes for all nine species. Full chloroplasts unambiguously differentiated all nine species, compared with the very few single nucleotide polymorphisms (SNPs) available with core DNA barcoding markers. SNPs for any two *Echinacea* chloroplast genomes ranged from 181 to 910, and provided robust data for unambiguous species delimitation. Implications for DNA-based species identification assays derived from chloroplast genome sequences are discussed in light of product safety, adulteration and quality issues.

## Introduction

*Echinacea*, i.e., purple coneflower, is one of the most popular botanicals used in dietary supplements. The range of *Echinacea* spans the Atlantic drainage region of the United States and extends into south central Canada^1^. For this genus, the Southern United Stated is an important native area with two species, i.e. *E*. *tennesseensis* and *E*. *laevigata* endemic to the southeast United States. Use of *Echinacea* products has dramatically increased: sales in 2013 increased by 94.7% over those in 2012, making it the 8^th^ most commonly sold herb in the United States^2^. By 2014, sales of *Echinacea* had increased by 79% from 2013 and it was the 3^rd^ most commonly sold herb in the United States with the sales surpassing $50 million^3^. Although not approved as a drug by the Food and Drug Administration, *Echinacea* products are often marketed for treatment of upper respiratory infections^4, 5^; other marketed uses include immune system stimulant^6, 7^, adjunct therapy for chronic candidiasis in women, and external wound healing^8^. Native Americans have been using *Echinacea* extensively to treat stomach cramps, rabies, toothaches, soremouth, throat, dyspepsia, colds, headache and snake bites^9^.

The three species used most commonly in dietary supplements are *E*. *purpurea*, *E*. *angustifolia* and *E*. *pallida*, available as teas, capsules and tablets. Importantly, each species appears to have different pharmacological activities, depending on the particular method of preparation and on which part of a given plant is used^8^. In addition to the three species, there are six other closely-related species in the same genus, i.e., *E*. *sanguinea*, *E*. *tennessensis*, *E*. *paradoxa*, *E*. *atrorubens*, *E*. *laevigata*, and *E*. *speciosa* ^10^. Ardjommand-woelkart and Bauer (2016), among others, have noted that both *E*. *angustifolia* (whole plant) and *E*. *purpurea* (dry root) have been associated with allergic reactions^11–13^. However, aside from these few instances, there are no known drug interactions or side effects^8^ associated with the 9 species.

The increased use of *Echinacea* species has led to concerns about adulterated products^14^. One of the reasons is that a few *Echinacea* species are phenotypically similar so it is easy to misidentify them if not familiar with the morphological variations among them^10^. The most common adulteration of *Echinacea* is the substitution of the root of *Parthenium integrifolium* for *E*. *purpurea* ^15^. The American Herbal Pharmacopoeia Standard of Identity includes additional adulterants for *E*. *purpurea*: *Helianthus* spp., *Lespedeza capita*, *Eryngium aquaticum*, and *Rudbekia nitida* (http://www.herbal-ahp.org/documents/macroscopy/Ech_purpurea_macro.pdf, accessed 09/13/16). Even when *Echinacea* species are being used in products, it is not easy to differentiate among the three most appropriate *Echinacea* species, i.e., *E*. *purpurea*, *E*. *angustifolia*, and *E*. *pallida*; as a result, mislabeling occurs frequently^15, 16^. Given that different species may enact different effects, such adulteration could decrease the safety, efficacy and reliability of commercial *Echinacea* products.

Distinguishing among *Echinacea* species using molecular methods is challenging due to extremely low levels of molecular divergence. This reflects a pattern seen among other members of Asteraceae, which demonstrate substantial morphological variation, but very little molecular differentiation, due to recent and rapid species radiations^17, 18^. Flagel *et al*.^19^ used three nuclear markers (*Adh*, *CesA*, and *GPAT*) and two plastid loci (*trnS* and *trnG*) to examine the phylogeny of *Echinacea*; however, no resolved topologies were obtained, suggesting incomplete lineage sorting, as well as the potential for widespread hybridization within the genus^19^.

DNA barcoding has been an effective tool for rapidly and accurately identifying many plant species^20–22^. Mitochondrial cytochrome c oxidase (*CO1*) has been successfully used as a barcode for animal species^23^; however, no single universal barcode has been entirely successful for distinguishing all plants to the species level^24^. In 2009, the Plant Working Group of the Consortium for the Barcode of Life (CBOL) proposed a 2-locus combination of *matK* + *rbcL* as a universal plant barcode; however, this approach only provides a discriminatory efficiency of 72%^20^. Many studies have shown that core DNA markers, i.e., *matK* and *rbcL*, cannot resolve closely-related species. For example, the commercially and medicinally important species of turmeric (*Curcuma longa*, Zingiberaceae) cannot be separated from almost a hundred other *Curcuma* species using *matK* and *rbcL* ^25^. A similar phenomenon was recently described for Venus slippers (*Paphiopedilum spp*.), where DNA barcodes were only successful 18.86% of the time for this popular family of orchids^26^. A study on DNA differentiation of pine nut samples conducted in our lab also indicated that the core barcoding markers were not effective for this group, so *ycf1* was developed for species level identification^27^.

Subsequently, two alternative strategies were proposed to discriminate among plant species: the first was the use of multiple loci^28–30^, and the second was the use of whole-chloroplast genomes, termed ‘super-barcoding’^31–34^. CBOL demonstrated that the use of seven plastid DNA barcoding markers only improved species discrimination from 72% to 73% when compared with the use of two core markers^20^. The idea of using whole chloroplast genomes to identify plant species was first proposed by Kane and Cronk (2008) and has been highlighted by a few recent review articles^22^. Using complete chloroplast genomes holds promise for efficient differentiation of species compared to a multi-locus approach, especially for closely related species such as *Echinacea*.

Advances in next-generation sequencing platforms have reduced the obstacles of time, effort, and cost, necessary to acquire whole chloroplast genomes. With earlier methods, chloroplast DNA had to be enriched, a time-consuming task requiring substantial fresh leaf tissue^35^. Approaches using polymerase chain reaction (PCR) enrichment, such as long PCR^36^ (using 27 primers) or multiple overlapping short-range PCR^37^ (using 138 primers), have been used, but these procedures are time-consuming and labor-intensive, and the primers used in such assays do not work equally well across different taxonomic groups. Nonetheless, complete chloroplast genomes have been shown to be highly effective for resolving relationships among species with low molecular divergence^32, 33, 38, 39^, and have been successfully employed for species identification^34^. Use of comparative chloroplast genomics has also been useful to identify divergent regions that can be employed for species-specific PCR-based diagnostics. For example, in 2013 Handy *et al*. used a large chloroplast dataset to design a species-specific assay to differentiate *Pinus armandii*, which causes a taste disturbance known as dysgeusia^40^, from other species that do not.

Although direct sequencing of genomic DNA is still costly, quickly advancing Next Generation Sequencing (NGS) technologies may ultimately prove to be more cost effective and technically efficient than other (often more time consuming) approaches to full chloroplast sequencing. For example, using the Illumina Miseq and Hiseq (Illumina, San Diego), 2 × 300 and 2 × 250 bp reads (respectively) can be obtained with rapid throughput kits (~27 hours) yielding as much as 12 to15 Gb from a MiSeq and as much as 60 to 120 Gb from a Hiseq. It was estimated that less than 1 GB of whole-DNA short reads can be effectively assembled into a full chloroplast genome with 51x coverage^41^. Therefore, this approach alleviates the need for expensive enrichment methods and fully leverages advances in DNA sequencing and bioinformatics.

In this study we extracted DNA from dried herbarium tissue samples for all 9 *Echinacea* species, sequenced each using the Illumina MiSeq platform, and here present complete chloroplast genomes for each species. Additionally, we highlight how variation within chloroplast regions can be utilized to develop rapid species-specific assays.

## Results

The data gathered for each species ranged from 434 MB for *E*. *tennessensis* to 2,531 MB for *E*. *purpurea*, with coverage of chloroplast genomes ranging between 20x for *E*. *tennessensis* and 65x for *E*. *angustifolia*. Additional information, including GenBank accession numbers, is available in Table 1.

**Table 1:** The nine species sampled in this study and information on the chloroplast genome assembly.

Species	Raw data size (MB)	Number of reads	Size of reads (bp)	Coverage of chloroplaste genome	Size of chloroplast genome (bp)	Accession number
*E*. *purpurea*	2,531	10,394,828	2 × 300	40	151,913	KX548224
*E*. *sanguinea*	2,437	10,966,208	2 × 250	51	151,926	KX548225
*E*. *tennessensis*	434	1,814,356	2 × 250	20	151,877	KX548223
*E*. *pallida*	832	4,078,614	2 × 250	33	151,883	KX548218
*E*. *paradoxa*	1,692	6,202,480	2 × 300	51	151,837	KX548217
*E*. *atrorubens*	472	1,923,846	2 × 250	31	151,912	KX548220
*E*. *laevigata*	545	2,198,622	2 × 250	28	151,886	KX548219
*E*. *angustifolia*	878	3,338,742	2 × 300	65	151,935	KX548221
*E*. *speciosa*	483	1,941,430	2 × 250	22	151,860	KX548222

**Figure 1:**
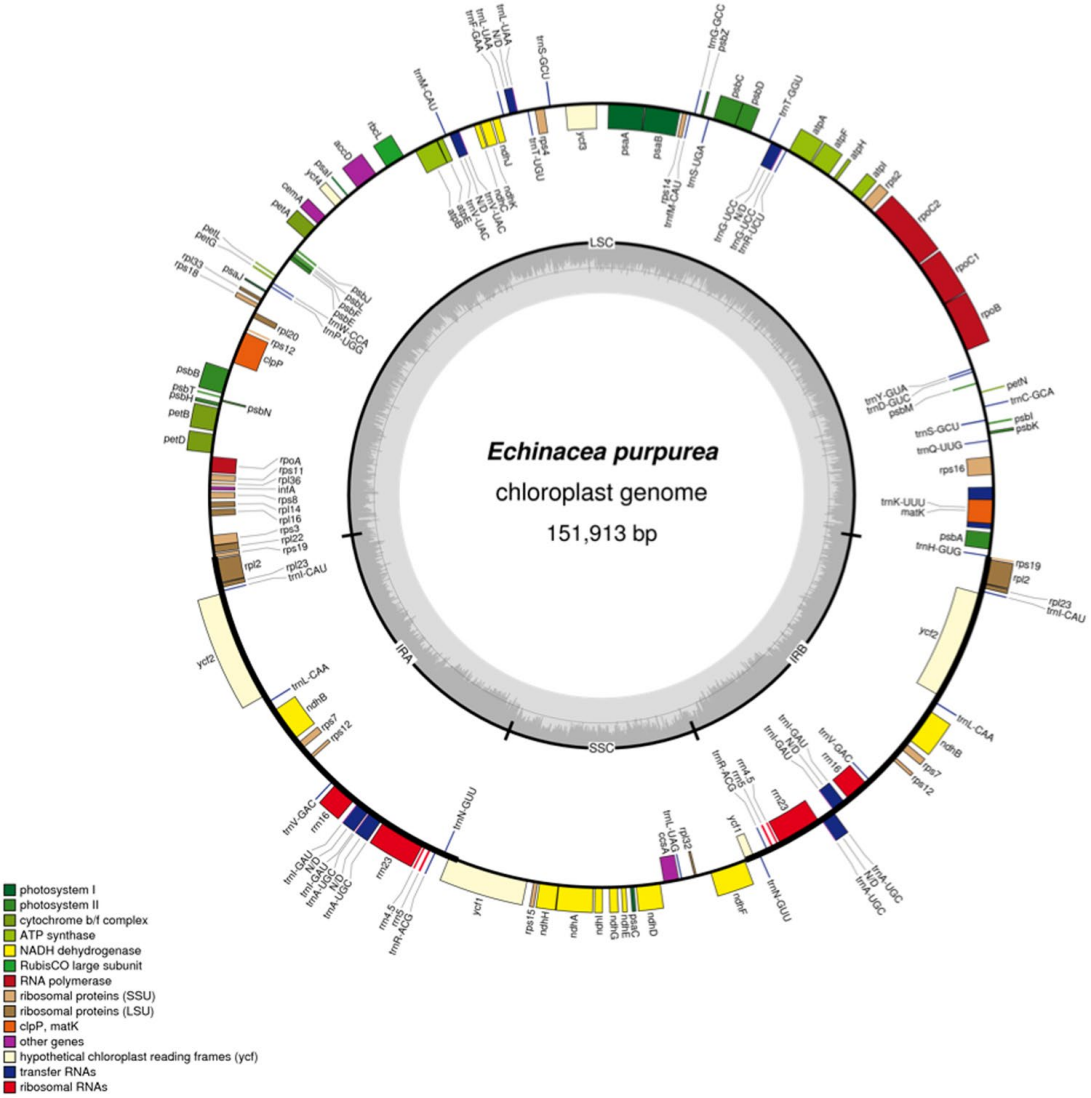
Gene map of the *Echinacea purpurea* chloroplast genome. Genes shown outside the circle are transcribed clockwise and those inside are transcribed counterclockwise. Gene belonging to different functional groups are color-coded as indicated by icons on the lower left corner. Dashed area in the inner circle indicates the GC content of the chloroplast genome. LSC, SSC and IR means large single copy, small single copy and inverted repeat, respectively.

The chloroplast genome of each *Echinacea* species appears to be collinear with the one of *Parthenium argentatum*, the most closely related public cpDNA genome, except for two inversions. These two inversions are specific to *P*. *argentatum* when compared with the other three Asteraceae species, i.e., *E*. *purpurea*, *Helianthus annuus*, and *Chrysanthemum indicum* (Figure S1). The first inversion is 891 bp long, located between *trnS* and *psbM*, and the second is 886 bp long, located between *psbM* and *rpoB*, these regions can be used for differentiating *P*. *argentatum* using PCR. In addition, positions of these two inversions in *Echinacea* species exchange with each other (Figure S1). Based on our alignments, no structural variations were detected among the nine *Echinacea* chloroplast genomes, so *E*. *purpurea* was used as an example to demonstrate the structure of *Echinacea* spp chloroplasts (Fig. 1).

The length of the chloroplast genome of *E*. *purpurea* is 151,913 bp. There are two inverted repeats (IRs) of 25,070 bp each, separated by a large single-copy and small single-copy (LSC and SSC) region of 83,602 bp and 18,171 bp, respectively. The G + C content of *E*. *purpurea* is 37.6% across the whole chloroplast genome. In total, there are 131 genes with 81 unique protein-coding genes, six of which are duplicated in the IR (Fig. 1). There are 18 unique genes with introns, five of which are duplicated in the IR; two genes have two introns and 16 genes have only one intron. There are 36 tRNA genes, 29 of which are unique and seven of which are duplicated in the IR. There are four unique ribosomal DNA and all of them are duplicated in the IR so there are eight ribosomal DNA in total.

As shown in Table 2, the number of base differences among these nine *Echinacea* species ranges from 181 (0.12%, *E*. *paradox* vs. *E*. *atrorubens*) to 910 (0.60%, *E*. *atrorubens* vs. *E*. *purpurea*). The number of differences between protein-coding genes is very low: 42 of 81 gene alignments are identical and the most divergent gene is *ycf1*, which has 31 variable sites and 4 indels within the 5059-bp alignment (Table 3). Table 4 lists the twenty-five most variable non-coding regions based on percentage of sequence identities. Eleven of these twenty-five overlap with those identified by Timme *et al*.^42^ and three overlap with the ten plastid markers proposed by Shaw *et al*.^43^ for low-level phylogenetic inferences^43^ (Table 4).

**Table 2:** Number and percentage of differences among nine *Echinacea* chloroplast genomes.

	*paradox*	*atrorubens*	*sanguinea*	*pallida*	*angustifolia*	*tennesseensis*	*laevigata*	*speciosa*	*purpurea*
*paradox*		0.12%	0.23%	0.18%	0.44%	0.52%	0.51%	0.50%	0.56%
*atrorubens*	181		0.20%	0.18%	0.48%	0.55%	0.55%	0.55%	0.60%
*sanguinea*	345	308		0.16%	0.45%	0.54%	0.53%	0.54%	0.60%
*pallida*	273	276	247		0.41%	0.50%	0.50%	0.50%	0.55%
*angustifolia*	672	727	685	629		0.47%	0.45%	0.45%	0.53%
*tennesseensis*	787	837	827	765	711		0.29%	0.20%	0.31%
*laevigata*	772	835	813	764	677	445		0.24%	0.31%
*speciosa*	768	830	827	767	689	309	365		0.23%
*purpurea*	849	910	908	842	811	469	478	350

**Table 3:** The 10 most-divergent coding regions among nine *Echinacea* species.

Genes	Length	Variable sites	Indels	Percentage of identical sites (%)	Timme *et al*.^42^
*ycf1*	5,049	31	4	99.0	√
*rps8*	405	3	0	99.3	
*rpoA*	1,009	4	1	99.3	
*rpoB*	3,198	7	1	99.3	
*petD*	483	3	0	99.4	
*matK*	1,282	6	0	99.4	√
*rbcL*	1458	7	0	99.5	
*ndhF*	2,232	11	0	99.5	√
*ndhI*	501	3	0	99.6	
*psbE*	252	1	0	99.6

**Table 4:** The 25 most-divergent non-coding regions among nine *Echinacea* species.

Genes	Length (bp)	Variable sites	Indels	Percentage of identical sites (%)	Timme *et al*.^42^	Shaw *et al*.^43^
*ccsA* → *trnL-UAG*	138	2	3	81.9		
*psbI* → *trnS-GCU*	144	4	5	86.8	√	
*5 S rRNA* → *trnR-ACG*	312	0	2	86.9		
*atpF* → *atpA*	72	0	2	88.9		
*rpl32* → *ndhF*	904	4	7	89.9	√	√
*trnT-UGU* → *trnL-UAA*	603	5	8	90.9	√	
*petN* → *psbM*	539	3	4	90.9	√	
*rps4* → *trnT-UGU*	392	3	3	91.6		
*petD* → *rpoA*	205	3	3	91.7		
*ndhI* → *ndhG*	388	3	1	92.5	√	
*trnT-GGU* → *psbD*	1270	11	8	92.9		√
*ndhD* → *ccsA*	234	2	4	93.2	√	
*trnH-GUG* → *psbA*	385	8	4	93.2	√	
*trnK-UUU* → *matK*	304	1	3	93.4	√	
*psbC* → *trnS-UGA*	246	1	3	93.6		
*ndhC* → *trnV-UAC*	998	9	7	93.9	√	√
*ycf3* → *trnS-GCU*	910	8	4	94.0	√	
*trnK-UUU* → *rps16*	783	2	5	94.1		
*trnR-UCU* → *trnG-UCC*	221	5	2	94.6	√	
*rps8* → *rpl14*	203	1	3	94.6		
*psaA* → *ycf3*	747	6	5	94.9		
*psaI* → *ycf4*	396	0	2	94.9		
*rpoC2* → *rps2*	259	0	2	95.0		
*rbcL* → *accD*	580	3	2	95.0		
*rps2* → *atpI*	233	1	1	95.3		

**Figure 2:**
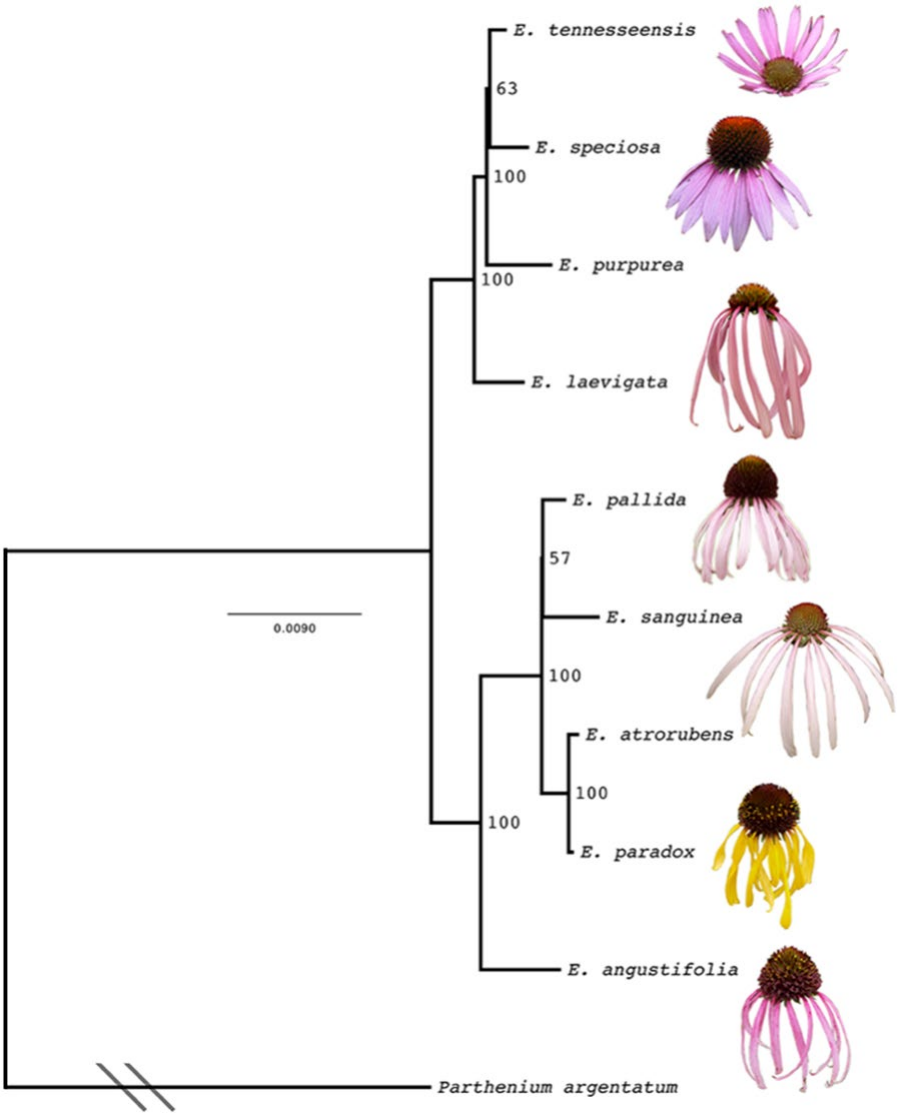
The ML tree of *Echinacea* reconstructed using chloroplast genomes. Numbers on branch nodes are bootstrap values. The branch connecting the outgroup *Parthenium argentatum* and nine *Echinacea* species was collapsed.

We used both coding and non-coding regions of the chloroplast genomes to effectively separate all *Echinacea* species and infer a phylogeny (Fig. 2). The nine *Echinacea* species separated into two clades with strong support. One clade is comprised of *E*. *tennesseensis*, *E*. *speciosa*, *E*. *purpurea* and *E*. *laevigata*. *E*. *tennesseensis* appears to be closely related to *E*. *speciosa* with a bootstrap value of 63%; and together they are both sister to *E*. *purpurea* with a bootstrap value of 100%. While *E*. *laevigata* is closely related to the other three species, i.e., *E*. *tennesseensis*, *E*. *speciosa*, and *E*. *purpurea*. The second clade is comprised of five species and is well-supported with a bootstrap value of 100%. *E*. *angustifolia* is closely related to the other four species, forming a clade with a bootstrap value of 100%. *E*. *atrorubens* is sister to *E*. *paradox* with a bootstrap value of 100%, and *E*. *pallida* is sister to *E*. *sanguinea* with a bootstrap value of 57%.

**Figure 3:**
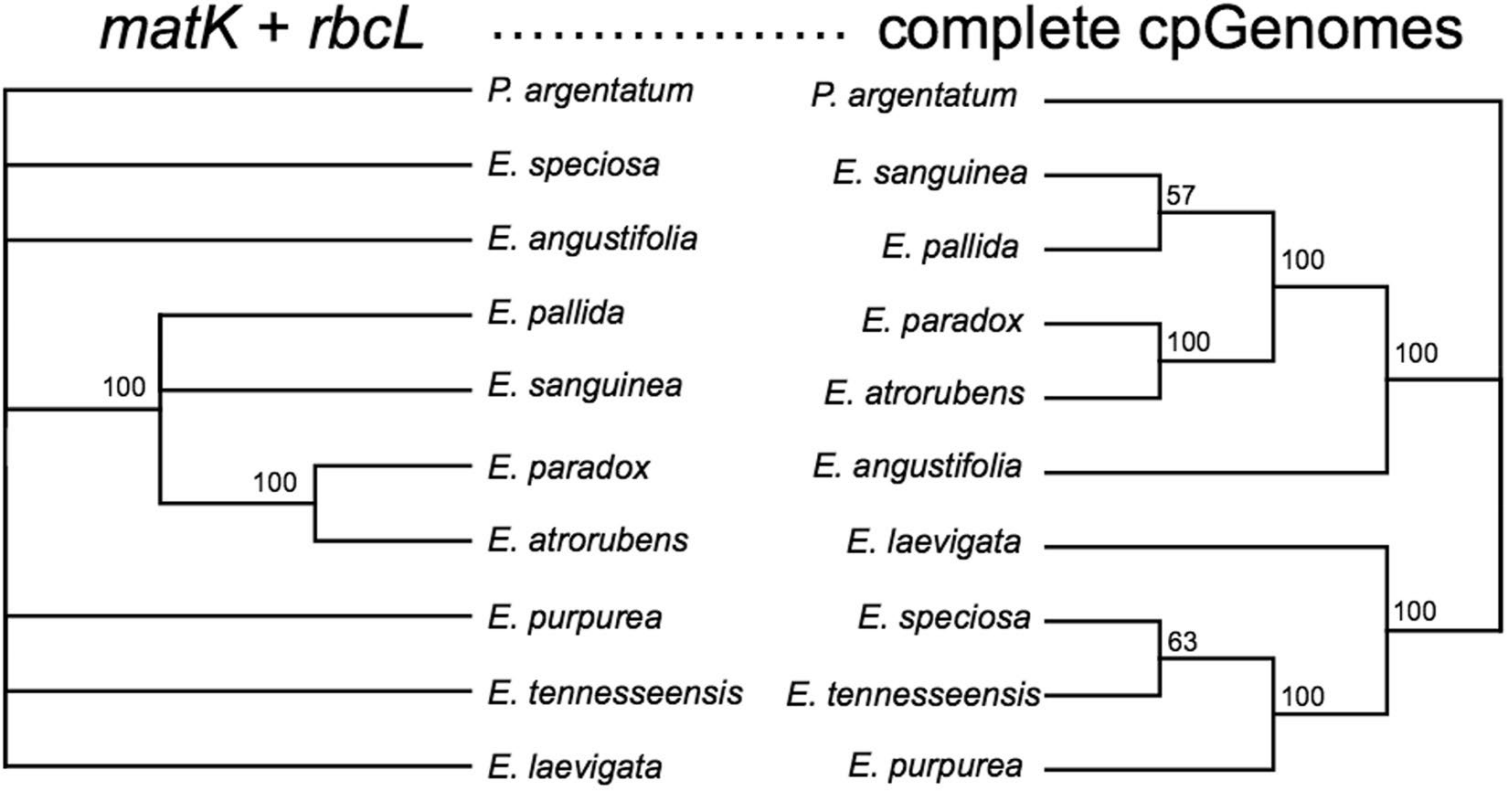
ML trees reconstructed using *matK* + *rbcL* (left) and using chloroplast genomes (right) Numbers are bootstrap values, branches with bootstrap values <50% are collapsed. These two phylogenies show the power of chloroplast genomes for delimitation of *Echinacea* species when compared with core DNA barcodes.

In contrast, using the core barcoding region *matK*, we only identified 5 variable sites and 0 variable sites for *rbcL* within the 943-bp and 599-bp alignments, respectively. Even using both markers, no variations between *E*. *purpurea* and *E*. *tennesseensis* or between *E*. *paradox* and *E*. *atrorubens* could be identified. As a result, the tree constructed using the two core DNA barcoding markers (*matK* and *rbcL*) provided no resolution at most nodes (Fig. 3). *E*. *pallida*, *E*. *sanguinea*, *E*. *paradox*, and *E*. *atrorubens* formed a clade with a bootstrap value of 100%, which is congruent with the one reconstructed using chloroplast genomes. *Echinacea paradox* is sister to *E*. *atrorubens* with a 100% bootstrap value. However, the positions of *E*. *pallida* and *E*. *sanguinea* were unresolved and the positions of the other five species could not be resolved using *matK* and *rbcL*. Therefore, these two core DNA markers are too conserved to use in diagnostic identification questions.

**Figure 4:**
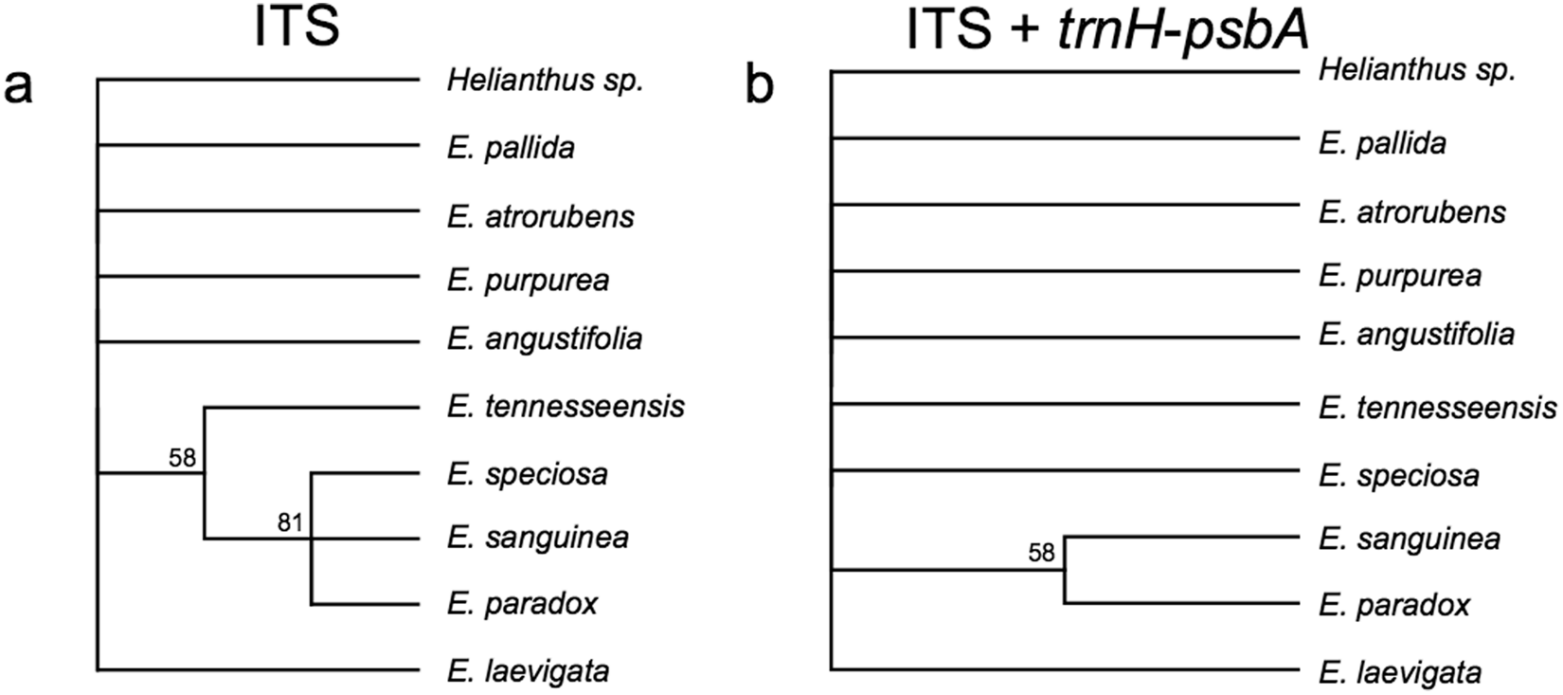
ML trees reconstructed using ITS (**a**) and ITS + *trnH*-*psbA* (**b**). Numbers are bootstrap values, branches with the bootstrap value <50% are collapsed. Both phylogenies show the lack of resolution among *Echinacea* species using either combination of genes.

Examination of the 727-bp alignment of ITS regions yielded only 7 variable sites. Additionally, no variation was observed among the three species: *E*. *atrorubens*, *E*. *purpurea*, and *E*. *angustifolia*. Thus, differentiation of *Echinacea* species using the ITS region was not robust. In the tree reconstructed using ITS, only 2 bootstrap values of 8 nodes were higher than 50% (Fig. 4a). *E*. *paradox*, *E*. *sanguinea*, and *E*. *speciosa* are highly supported as one clade with a 81% bootstrap value; *E*. *angustifolia*, *E*. *purpurea*, *E*. *atrorubens*, *E*. *laevigata*, and *E*. *pallida* group into one clade with a bootstrap value of 58%. Interestingly, the topology reconstructed using ITS is substantially different from the one obtained using chloroplast genomes (Fig. 3).

The alignment of the nine *Echinacea* chloroplast genomes suggests that the intergenic region between *trnH* and *psbA* may be an appropriate gene for DNA barcoding for the majority of *Echinacea* species - especially if used in combination with ITS. However, differentiation relies upon very few SNPs so validation using a greater number of authenticated individuals would be needed. The size of the *trnH*-*psbA* PCR product ranges from 499 (*E*. *purpurea*) to 511 bp (*E*. *laevigata*) and the number of SNPs between any two species ranges from 0 (*E*. *atrorubens* vs *E*. *paradox* and *E*. *speciosa* vs *E*. *tennesseensis*) to 16 (*E*. *laevigata* vs E. *purpurea*) (Table S1). According to the chloroplast alignment, universal primers for *trnH*-*psbA* (trnHf_05^44^/psbA3_f^45^) should successfully amplify all 9 *Echinacea* species. In addition, the alignment indicates that pairs of species that cannot be differentiated using *trnH*-*psbA* alone, such as (*E*. *atrorubens* and *E*. *paradox*a) and (*E*. *speciosa* vs *E*. *tennesseensis*) could in theory be differentiated with the addition of the *ITS* marker. However, even with both markers, the number of diagnostic SNPs ranges from only 1 (*E*. *speciosa* vs *E*. *tennesseensis*) to 18 (*E*. *purpurea* vs *E*. *laevigata*) (Table S2) and bootstrap values for the tree constructed with *trnH* and *psbA and ITS* are extremely low (Fig. 4b).

## Discussion

We successfully used direct sequencing of genomic DNA to recover complete chloroplast genomes from all nine reported *Echinacea* species and demonstrated that full chloroplast genomes can effectively differentiate all nine species. In addition to clarifying relationships among species, chloroplast genomes provide valuable data for improved DNA-based identification assays. This is especially true for closely related species, such as *Echinacea* that cannot be currently identified using most core DNA barcoding markers.

Conclusive documentation of indels could identify regions for use with PCR based screening diagnostics^46^. For example if a region that distinguishes important species based on the size of DNA fragments can be identified and validated, this method could be used without sequencing, thus creating a rapid low cost approach to species identification. In the absence of suitable indels, other variable regions in closely related species can be targeted for either PCR, real-time PCR or other sequence based identification methods^40^.

There are currently 916 chloroplast genomes of land plants available in GenBank, among them, 456 (49.8%) were sequenced since 2015. With the advancement of NGS technologies and bioinformatics tools, obtaining chloroplast genomes has become quick and relatively inexpensive. Some methods developed for metagenomics, like kSNP^47^, Kraken^48^ and Pathoscope^49^, can be used to identify species using whole-genome sequencing data in conjunction with genome scale references. We are currently investigating these options, and they will be the focus of a future manuscript.

The data generated for this *Echinacea* inquiry will become part of the U.S. Food and Drug Administration’s library of chloroplast genomes, the details of which will be discussed in a future publication. Future studies will explore the most useful and efficient way to identify *Echinacea* species using either whole chloroplast genomes or targeted assays developed from the full chloroplast genomes.

## Methods

### Sampling

We sampled all nine *Echinacea* species available from the U.S. National Herbarium. Voucher information can be found in Table 1 and Table 5.

**Table 5:** Sampling in this *Echinacea* study.

Species	Voucher	Year collected
*E*. *purpurea*	US 2349097	1958
*E*. *sanguinea*	US 1468035	1930
*E*. *tennessensis*	US 980416	1916
*E*. *pallida*	US 2233063	1948
*E*. *paradoxa*	US 1653013	1935
*E*. *atrorubens*	US 2235164	1955
*E*. *laevigata*	US 3360860	1998
*E*. *angustifolia*	US 2802433	1974
*E*. *speciosa*	US 2349080	1960

### DNA isolation, and sequencing

Total DNA was extracted from the dry leaves of specimens using the DNeasy Plant Mini Kit (part #69106, Qiagen, Valencia, CA,). For the library construction, 200 ng DNA was taken and sheared into ~550 bp contigs with the Covaris M220 Focused-ultrasonicator. The library was constructed using either the TruSeq DNA HT Sample Prep Kit (Illumina, FC-121-3003) or the TruSeq Nano DNA NeoPrep Kit (Illumina, NP-101-1001). Sequencing was run on the Illumina MiSeq Sequencer with MiSeq Reagent Kit v2 (MS-102-2001) or MiSeq Reagent Kit v3 (MS-102-3001) to obtain 2 × 250 or 2 × 300 reads, respectively.

### Genome assembly and annotation

Before assembly, the reads were trimmed using the Qiagen CLC Genomics Workbench v.8.5.1 (hereafter called CLC) with default settings. Then the trimmed sequences were assembled into contigs using *de novo* assembly, implemented in CLC. In addition, a reference-guided assembly was performed using CLC with the published chloroplast genome of the closest available relative, *Parthenium argentatum* (NC_013553), as the reference genome. After finishing reference-guided assembly, a consensus sequence of *Echinacea* was obtained. Both the consensus sequence from the reference-guided assembly and the contigs from the *de novo* assembly were imported into Geneious Pro 9.0.4, and then those contigs of chloroplast were mapped onto the consensus sequence. The mapped contigs were checked and adjusted manually to align with the consensus sequence obtained using referenced-guided assembly^39^. The final sequence of *Echinacea* chloroplast genome is the ordered sequence of those mapped contigs. We annotated the chloroplast genome using Geneious with the chloroplast genome of *Helianthus annuus* (NC_007977) as the reference since the annotation of *H*. *annuus* is known to be accurate^42, 50^. All sequence data has been deposited in Genbank (Accession numbers KX548217- KX548225, Table 1).

### Retrieving gene sequences of widely-used DNA barcoding markers

In order to test if core DNA barcode markers can be used for identification here, we obtained gene sequences of *matK*, *rbcL*, and ITS (internal transcribed spacer) for *Echinacea* species and for their closely-related species. In order to be effective, these needed to have variable bases in each of the nine species being investigated.

Based on the alignment of *P*. *argentatum* with nine *Echinacea* chloroplast genomes, we extracted two core plastid DNA barcoding markers *matK* and *rbcL*. These markers used for DNA barcoding were delimitated by corresponding primers, rbcLa-F (ATGTCACCACAAACAGAGACTAAAGC)^51^/rbcLa-R (GTAAAATCAAGTCCACCRCG)^28^ for *rbcL*, matK-xf (TAATTTACGATCAATTCATTC)^52^/matK-MALP (ACAAGAAAGTCGAAGTAT)^53^ for *matK*.

We also obtained the gene sequences of ITS, another commonly used marker, from each *Echinacea* species. To obtain the ITS sequence for each species, the contig containing the ITS was obtained. The contigs of each species obtained using *de novo* assembly mentioned above were built into a BLAST database on the local server, then the ITS sequence of *Echinacea pallida* (EU785938) was used as the seed to search against the database. Usually, the best-hit contig contains the sequence of ETS, 18S, ITS1, 5.8S, ITS2, and 26S. Then we delimitated the region of ITS using the corresponding primers, i.e., ITS1 (TCCGTAGGTGAACCTGCGG)^54^/ITS4 (TCCTCCGCTTATTGATATGC)^54^. Since the ITS sequence of *P*. *argentatum* is not available, *H*. *annuus* (JX867644) was used as the outgroup.

### Phylogenetic analysis

Whole chloroplast genomes of nine *Echinacea* species and the one of *Parthenium* were aligned using MAFFT v7^55^. As the sequences of IRa and IRb are almost identical, only one of them was included in the phylogenetic analyses. In addition, the sequences of tRNAs and rDNAs of nine *Echinacea* species are almost identical, so those genes were removed for all samples from the alignment. In order to reduce phylogenetic noise, three inverted intergenic regions of *Parthenium* were deleted from the alignment. The program PartitionFinder^56^ was used for identifying partitions used in developing model parameters for phylogeny estimation. A maximum likelihood (ML) tree was inferred with RAxML v8.1^57^ using the model of GTRGAMMAI, and 1,000 rapid bootstrap replications were performed. The sequences of *matK* + *rbcL* and ITS were aligned with MAFFT v7, then the ML trees were reconstructed using RAxML with the GTRGAMMAI model, and 1,000 rapid bootstrap replications were performed. Since this study mainly focuses on species delimitation rather than phylogeny, these genes were not concatenated for further phylogenetic analyses. These alignments were deposited into the DRYAD with the accession number of XXXX.

## Electronic supplementary material

Supplementary Information
