# Supplementary material for: An analysis of Echinacea chloroplast genomes: Implications for future botanical identification
Source: Sci Rep. 2017 Mar 16;7:216. doi: 10.1038/s41598-017-00321-6 (PMC5428300; doi:10.1038/s41598-017-00321-6)
Supplement: Supplementary file 1 — Supplementary Information [file 41598_2017_321_MOESM1_ESM.pdf]

An analysis of *Echinacea* chloroplast genomes: Implications for future botanical identification

Ning Zhang[1,*], David L. Erickson[1], Padmini Ramachandran[1], Andrea R. Ottesen[1], Ruth E. Timme[1], Vicki A. Funk[2], Yan Luo[1], Sara M. Handy[1]

Figure S1. Structural variation among cpGenomes of several Asteraceae species. Two inversions that differentiate *Parthenium argentatum* from the other three species were observed, although the *Echinacea* clade was still more closely related to *Parthenium* than the other Asteraceae despite the inversion. Such structural inversions can be used in development of diagnostic PCR assays that differentiate among these closely related species.

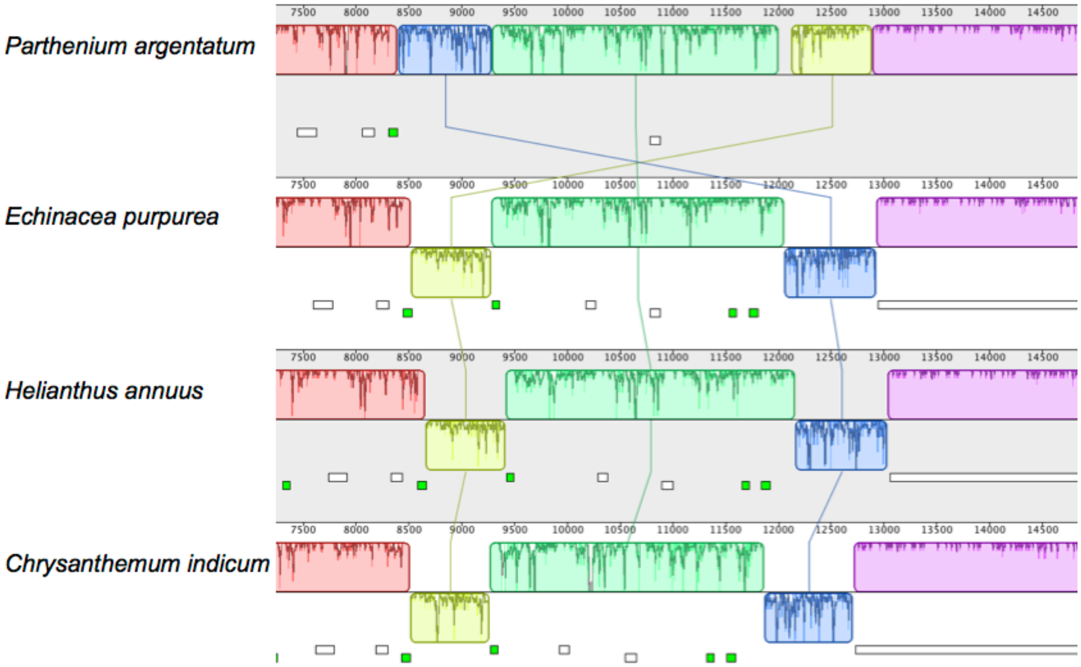

Table S1, Number of differences among nine *Echinacea trnH-psbA*.

	paradox	*atrorubens*	*sanguinea*	*pallida*	*angustifolia*	*tennesseensis*	*laevigata*	*speciosa*	*purpurea*
paradox		0	5	2	7	5	11	5	13
atrorubens	0		5	2	7	5	11	5	13
sanguinea	5	5		3	8	6	10	6	14
pallida	2	2	3		5	3	9	3	11
angustifolia	7	7	8	5		4	10	4	12
tennesseensis	5	5	6	3	4		8	0	10
laevigata	11	11	10	9	10	8		8	16
speciosa	5	5	6	3	4	0	8		10
purpurea	13	13	14	11	12	10	16	10	

Table S2, Number of differences among nine *Echinacea trnH-psbA* + ITS

	paradox	*atrorubens*	*sanguinea*	*pallida*	*angustifolia*	*tennesseensis*	*laevigata*	*speciosa*	*purpurea*
paradox		3	6	6	10	7	16	6	16
atrorubens	3		8	3	7	6	13	7	13
sanguinea	6	8		7	11	8	14	7	17
pallida	6	3	7		6	5	12	6	12
angustifolia	10	7	11	6		5	12	6	12
tennesseensis	7	6	8	5	5		11	1	11
laevigata	16	13	14	12	12	11		12	18
speciosa	6	7	7	6	6	1	12		12
purpurea	16	13	17	12	12	11	18	12	
